# Three-Dimensional Thermal Tomography with Physics-Informed Neural Networks

**DOI:** 10.3390/tomography10120140

**Published:** 2024-11-30

**Authors:** Theodoros Leontiou, Anna Frixou, Marios Charalambides, Efstathios Stiliaris, Costas N. Papanicolas, Sofia Nikolaidou, Antonis Papadakis

**Affiliations:** 1 Department of Mechanical Engineering, Frederick University, Nicosia 1036, Cyprus; 2 Computation-Based Science and Technology Research Center (CaSToRC), The Cyprus Institute, 20 Kavafi Street, Nicosia 2121, Cyprus; a.frixou@cyi.ac.cy (A.F.); stiliaris@phys.uoa.gr (E.S.); c.papanicolas@cyi.ac.cy (C.N.P.); 3 Department of Business Administration, Frederick University, Nicosia 1036, Cyprus; bus.chm@frederick.ac.cy; 4 Department of Physics, National and Kapodistrian University of Athens, Zografou, GR-157 84 Athens, Greece; 5 KYAMOS Ltd., 37 Polyneikis Street, Strovolos, Nicosia 2047, Cyprus; snikolaidou@kyamosmultiphysics.com (S.N.); apapadakis@kyamosmultiphysics.com (A.P.); 6 Department of Electrical, Computer Engineering and Informatics, Frederick University, Nicosia 1036, Cyprus

**Keywords:** thermal tomography, convolutional neural networks, physics-informed neural networks, 3D temperature field, heat conduction, inverse problems, non-destructive testing

## Abstract

**Background**: Accurate reconstruction of internal temperature fields from surface temperature data is critical for applications such as non-invasive thermal imaging, particularly in scenarios involving small temperature gradients, like those in the human body. **Methods**: In this study, we employed 3D convolutional neural networks (CNNs) to predict internal temperature fields. The network’s performance was evaluated under both ideal and non-ideal conditions, incorporating noise and background temperature variations. A physics-informed loss function embedding the heat equation was used in conjunction with statistical uncertainty during training to simulate realistic scenarios. **Results**: The CNN achieved high accuracy for small phantoms (e.g., 10 cm in diameter). However, under non-ideal conditions, the network’s predictive capacity diminished in larger domains, particularly in regions distant from the surface. The introduction of physical constraints in the training processes improved the model’s robustness in noisy environments, enabling accurate reconstruction of hot-spots in deeper regions where traditional CNNs struggled. **Conclusions**: Combining deep learning with physical constraints provides a robust framework for non-invasive thermal imaging and other applications requiring high-precision temperature field reconstruction, particularly under non-ideal conditions.

## 1. Introduction

Thermal tomography is a non-invasive imaging technique that reconstructs three-dimensional images of an object’s internal temperature distribution by compiling cross-sectional images layer by layer or slice by slice [1,2,3,4,5,6,7,8,9,10]. This methodology relies on temperature or heat transfer measurements collected from the object’s surface [4], and it has gained significant attention due to its potential applications in non-destructive testing, material characterization, and medical diagnostics [9,10,11,12]. The primary objective of this technique is to detect internal heat sources or variations in thermal properties by solving inverse problems associated with surface temperature measurements [13,14].

Thermography, and in general thermal imaging, has shown promise in several engineering applications. In material science, thermography was proved to detect defects in composite materials [15,16] and hidden corrosion in metals [17] and assess delamination in ceramic thermal coatings [1,18]. For instance, thermal imaging has been effectively utilized to detect the delamination of ceramic thermal barrier coatings in high-temperature turbine blades [19,20]. Another study by Curran et al. [21] demonstrated that tomographic reconstructions enhance the accuracy of a combined radiation and conduction model for predicting thermal conductivity in ceramic fiber insulation.

Beyond material science, two-dimensional (2D) passive infrared thermography is widely used to identify temperature irregularities in non-destructive testing [22,23], in the food industry [24], in biological observation [25], and in medical applications [26]. In medical applications, thermal imaging can detect various conditions by analyzing temperature distributions within the human body. For example, Kaczmarek and Nowakowski [27] demonstrated the use of active dynamic thermography (ADT) for monitoring skin burn healing and post-surgical wounds. This study emphasizes the benefits of combining quantitative thermal data with traditional imaging methods, supporting a multimodal approach to enhance diagnostic accuracy. Furthermore, abnormal heat distributions captured by infrared thermal (IR) images may indicate underlying pathologies such as tumors, inflammations, or circulatory abnormalities [3]. Various algorithms have been developed for the analysis of 2D planar thermal images in diagnosing breast cancer [28].

The first attempt to go beyond 2D thermography and reconstruct 3D tomographic images with low-temperature hot-spots was performed by Koutsantonis et al. using RISE methodology [6], followed by the work of Ledwon et al. [3] and Sage et al. [5] using convolutional neural networks (CNNs). Unlike infrared thermography, which relies on surface temperature data, thermal tomography is a volumetric imaging technique that aims to understand the thermal properties within the whole volume, retaining spatial information and identifying subsurface anomalies [4]. Extending these capabilities to three-dimensional (3D) thermal tomography offers a more comprehensive tool for non-invasive imaging [5]. Recent experimental results with hardware phantoms suggest that thermal tomography may be feasible even for challenging cases of low-temperature hot-spots (1–4 °C) at depths reaching 1.5 cm below the skin [29]. Additionally, Kaczmarek and Nowakowski [27] further highlighted the benefits of combining quantitative thermal data with traditional imaging methods for more accurate diagnostics.

### 1.1. Convolutional Neural Networks (CNNs)

Recent developments in machine learning, particularly convolutional neural networks (CNNs), have significantly enhanced the capabilities of thermal tomography. CNNs have been successfully applied to reconstruct temperature distributions from planar thermal images, demonstrating improved accuracy over traditional reconstruction methods such as algebraic reconstruction techniques (ART) and maximum-likelihood expectation-maximization (MLEM) [7]. These advancements have opened new avenues for utilizing deep learning models in thermal imaging, allowing for more accurate reconstructions even in the presence of complex internal structures and multiple heat sources [2].

Several studies have demonstrated the capability of CNNs to enhance thermal tomography performance. In one such study, Ledwon et al. [3] applied CNNs to reconstruct thermal fields from surface temperature data, showing improved accuracy over conventional methods like algebraic reconstruction techniques (ART). Their model could handle complex geometries and irregularities in the temperature field, making it suitable for real-world applications in non-destructive testing. Similarly, Sage et al. [5] utilized CNNs to tackle the inverse problem of reconstructing internal temperature fields, particularly in scenarios involving low-temperature contrast, which are typical in biological tissues. The use of CNNs enabled faster and more accurate reconstructions compared to iterative solvers.

Despite these advances, CNNs still face limitations when predicting temperature fields in larger domains or deeper regions where surface data become less informative. Yao et al. [30] highlighted this challenge, showing that CNN-based models struggled to accurately reconstruct internal temperatures in larger volumes, particularly when surface measurements were noisy. This has spurred interest in hybrid approaches that combine CNNs with physics-based models to address the limitations of purely data-driven methods.

### 1.2. Physics-Informed Neural Networks (PINNs)

Physics-informed neural networks (PINNs) are a novel approach that embed physical laws, typically expressed as partial differential equations (PDEs), directly into the training process of neural networks. By leveraging governing equations, such as the heat equation or Navier–Stokes equations, PINNs enforce consistency between predicted and expected solutions, making them particularly effective for ill-posed inverse problems. Previous work suggests that PINNs can significantly enhance predictions in scenarios where adherence to physical laws is crucial for accuracy. For example, Tod et al. [31] employed PINNs to improve physics-based models for predicting the thermal behavior of stereolithography (SLA) processes, comparing predicted temperature fields with measurements obtained from high-speed infrared thermal cameras. Similarly, Bhatnagar et al. [10] applied PINNs to solve the 3D incompressible Navier–Stokes equations at moderate to high Reynolds numbers using sparsely distributed solution data, demonstrating their effectiveness in surrogate modeling for design problems governed by nonlinear PDEs. Kim et al. [32] provide a comprehensive review of recent advancements in PINNs, emphasizing their ability to solve inverse problems in scenarios with sparse, noisy, or incomplete data. These methods have been applied across diverse fields, including fluid dynamics [33], biomedical imaging [34], and material characterization [35], showcasing their versatility and robustness. Despite their promise, challenges remain in terms of computational efficiency and scalability [36], which require further investigation to enhance their applicability to real-world problems.

### 1.3. Contributions and Novelty

In this study, we build upon established deep learning techniques to enhance thermal tomography, utilizing a dataset containing phantoms with multiple low-temperature regions similar to those encountered in the human body. The input to our model consists of a representation of surface temperature distributions, which are processed through a 3D convolutional neural network (CNN) to achieve accurate 3D reconstructions of internal temperature fields. We examine both ideal conditions, without noise or systematic errors, and more realistic scenarios accounting for sensor inaccuracies and background temperature fluctuations.

This study investigates the use of a hybrid framework combining 3D convolutional neural networks (CNNs) and physics-informed neural networks (PINNs) for thermal tomography. While previous works have demonstrated the potential of CNNs for reconstructing internal temperature fields, these approaches often face challenges when applied to noisy data or larger domains where surface measurements are less informative. Our approach incorporates the laws of heat conduction directly into the learning process, aiming to address these challenges and improve prediction accuracy and robustness.

The key contributions of this work are as follows:

1. Integration of PINNs with CNNs: By embedding physical principles into the CNN training process, we aim to improve prediction robustness, particularly under non-ideal conditions with noise and background temperature variations.
2. Exploration of larger domains: We evaluate the framework’s performance across a range of domain sizes, highlighting its potential for predicting deeper temperature fields while discussing its limitations.
3. Improved robustness to noise: Through noise-aware training and physics-informed loss functions, we demonstrate enhanced reconstruction accuracy in scenarios where purely data-driven approaches struggle.

These contributions provide insights into how hybrid methods can complement traditional data-driven techniques. While preliminary, this work offers a pathway to improving thermal imaging applications such as medical diagnostics and non-destructive testing.

## 2. Materials and Methods

### 2.1. The Training Dataset

To generate synthetic data for thermal tomography, we solve the heat equation within a cylindrical region representing a phantom. The phantom consists of a medium with conductivity similar to that of the human body, containing cylindrical hotspots, which simulate regions of varying thermal activity. The heat equation in three dimensions is given by:

(1) $$\frac{\partial T(x,y,z,t)}{\partial t}=\kappa \nabla^{2}T\left(x,y,z,t\right)+\frac{S(x,y,z)}{\rho c},$$

where T(x,y,z,t) represents the temperature at any point (x,y,z) and time *t*, $\kappa =\frac{k}{\rho c}$ is the thermal diffusivity, *k* is the thermal conductivity, ρ is the mass density, *c* is the specific heat capacity, and S(x,y,z) is the thermal source term. The source term S(x,y,z) is non-zero only at locations corresponding to the hotspots.

In our study, we focus on the steady-state solution where the time derivative of temperature is zero (∂T/∂t=0). This reduces the heat equation to a Poisson equation:

(2) $$\nabla^{2}T\left(x,y,z\right)=-\frac{S(x,y,z)}{k}.$$

The equation is discretized using finite differences on a 3D rectangular grid, leading to a system of linear equations that can be solved iteratively. The Jacobi method is employed for solving this discretized system, where the temperature at each grid point is updated iteratively based on the average temperature of its neighboring points and the source term.

The boundary conditions applied to the cylindrical phantom are convective boundary conditions. At the boundary, the heat flux is proportional to the temperature difference between the surface of the phantom and the surrounding ambient air:

(3) $$k\frac{\partial T_{s}}{\partial r}=h\left(T_{s}-T_{\infty }\right),$$

where $T_{s}$ is the surface temperature of the phantom, $T_{\infty }$ is the ambient temperature, *h* is the convective heat transfer coefficient, and *k* is the thermal conductivity of the gel. The value of the thermal conductivity of the medium was chosen to be 0.5 W/(mk) and the heat convection (used in the boundary condition-cooling from air) = 7.5 W/(m^2^K).

**Figure 1:**
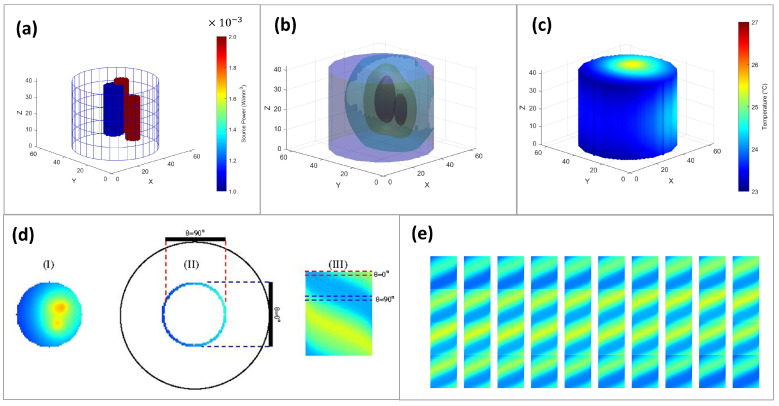
(**a**) The sources used for the heat equation. In this example 3 cylindrical sources of various sizes have been used located within the cylindrical region. (**b**) The 3D temperature field resulting from the solution of the heat equation. The ambient air temperature is $22^{\circ }$. The solution in our case consists of 40 slices along the z-axis, each with dimensions 64×64. (**c**) The surface temperature. In principle, this is what can be observed by the detector. (**d**) The process of generating the input to the neural network. (I) The solution consists of 40 slices along the z-axis. The central slice is illustrated. (II) For each slice, the surface temperature is projected to the detector for a total of 64 different angles (projections) with values $0^{\circ }\leq \theta <360^{\circ }$. Here, the cases with $\theta =0^{\circ }$ and $\theta =90^{\circ }$ are illustrated with dotted lines indicating the region visible to the detector. (III) All the projections are collected to form the sinogram for the particular slice. (**e**) The collection of all sinograms (40 in this case) is used as input to the neural network, which uses them to predict the 3D temperature field.

Figure 1 illustrates the domain, sources, and the solution of the heat equation for a specific case of three cylindrical sources (Figure 1a). The simulation domain is discretized into a grid with dimensions 64 ×64×40 along the x-, y-, and z-axes, respectively, and solved up to a tolerance of 0.001 C (Figure 1b). From the entire solution, only the temperature field at the surface is used (Figure 1c), which is then projected to 2D space, creating thermal sinograms corresponding to the signal measured by the detector as is illustrated in parts (d) and (e) of Figure 1. The input to the neural network consists of 40 sinograms each with dimensions 64×64 (Figure 1e).

The synthetic dataset generated using this forward model consists of temperature distributions with up to six randomly distributed cylindrical hotspots of varying dimensions and power. We use phantoms with physical diameters ranging from 10 cm to 50 cm. The cylindrical sources have random heights and radii, where, for example, in the case of the 10 cm phantom the heights are in the range between 6 mm and 60 mm and the radii between 2 mm and 16 mm. There is no minimum distance between the sources, and they may even overlap and are placed anywhere within the phantom, at least 2 mm away from the boundary, but each source must fully fit.

A total of 6000 training solutions (1000 for each number of hotspots) were generated, then randomized and split into training, validation, and testing datasets with 4000, 1000, and 1000 members, respectively.

### 2.2. The Neural Network

**Figure 2:**
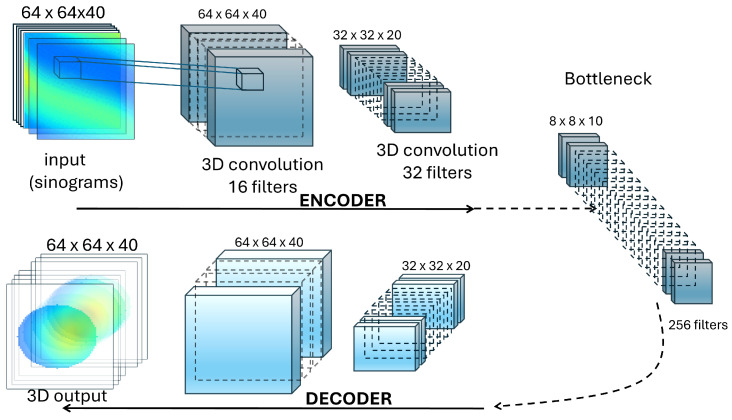
A simplified representation of the 3D convolutional autoencoder used. The input consists of all the sinograms (see description in Figure 1) which capture the surface temperature as seen by the detector. The output is the full 3D temperature field. For simplicity, not all layers are shown (denoted by the use of dashed arrows). Details about the number of layers, residual blocks, kernel sizes, and hyperparameters can be found in the text below.

The proposed neural network is a 3D convolutional autoencoder designed for the reconstruction of temperature fields in the form of 3D images. The network is structured in an encoder–decoder architecture, where the encoder compresses the input data into a latent representation, and the decoder reconstructs the data back to the original dimensions. A simplified representation is provided in Figure 2.

The encoder consists of multiple 3D convolutional layers followed by residual blocks to enhance feature extraction and maintain gradient flow. Specifically, each residual block is composed of two convolutional layers with batch normalization and ReLU activations. This setup helps the model to learn complex features while mitigating the vanishing gradient problem. The dimensionality of the data is reduced through strided convolutions, allowing the network to capture higher-level abstractions in the thermal data.

The decoder mirrors the encoder but uses transposed convolutions to upsample the latent representation back to the original resolution. Like the encoder, the decoder employs residual blocks to refine the reconstructed image, preserving the details and sharpness in the output.

In this study, we build upon established deep learning methods for thermal tomography, particularly utilizing 3D convolutions and residual blocks for enhanced reconstruction quality. Our model follows the principles outlined in previous works, such as those by Ledwon et al. [3], Toivanen et al. [4], and Sage et al. [5], adapting them for the reconstruction of 3D temperature fields from volumetric data. Rather than introducing novel techniques, we refine these approaches, applying them to our specific use case to achieve high-resolution thermal field reconstructions. Overall, the proposed autoencoder efficiently reconstructs high-resolution temperature fields from 3D thermal sinograms (input = planar images not tomographic; the third dimention is the angles), demonstrating its suitability for non-destructive thermal testing and defect detection applications.

The network was designed to predict the 3D temperature field with the same dimensions as the input, i.e., 64×64×40. The details of the CNN used are as follows:

**Encoder:**

- Input: The input tensor has dimensions 64×64×40×1.
- Layer 1: A 3D convolutional layer with 16 filters, kernel size 3×3×3, strides of 1×1×1, and ‘same’ padding. This is followed by a residual block with 16 filters.
- Layer 2: A 3D convolutional layer with 32 filters, kernel size 3×3×3, strides of 2×2×2, and ‘same’ padding. This downscales the input. This is followed by a residual block with 32 filters.
- Layer 3: A 3D convolutional layer with 64 filters, kernel size 3×3×3, strides of 2×2×2, and ‘same’ padding. This is followed by a residual block with 64 filters.
- Layer 4: A 3D convolutional layer with 128 filters, kernel size 3×3×3, strides of 2×2×1, and ‘same’ padding. This is followed by a residual block with 128 filters.
- Bottleneck: A 3D convolutional layer with 256 filters, kernel size 3×3×3, and ‘same’ padding, followed by a residual block with 256 filters.

**Decoder:**

- Layer 5: A 3D transposed convolutional layer with 128 filters, kernel size 3×3×3, strides of 2×2×1, and ‘same’ padding. This is followed by a residual block with 128 filters.
- Layer 6: A 3D transposed convolutional layer with 64 filters, kernel size 3×3×3, strides of 2×2×2, and ‘same’ padding. This is followed by a residual block with 64 filters.
- Layer 7: A 3D transposed convolutional layer with 32 filters, kernel size 3×3×3, strides of 2×2×2, and ‘same’ padding. This is followed by a residual block with 32 filters.
- Layer 8: A 3D transposed convolutional layer with 16 filters, kernel size 3×3×3, strides of 1×1×1, and ‘same’ padding. This is followed by a residual block with 16 filters.
- Output Layer: A 3D convolutional layer with 1 filter, kernel size 3×3×3, ‘same’ padding, and ReLU activation, providing the predicted temperature field with dimensions 64×64×40×1.

**Residual Block:** Each residual block consists of two 3D convolutional layers, each followed by batch normalization and ReLU activation. A skip connection adds the input of the block to the output of the second convolution, ensuring the flow of information from earlier layers and mitigating the vanishing gradient problem.

### 2.3. Computational Efficiency

One key consideration in deploying deep learning frameworks like the one proposed in this study is computational efficiency. While the training phase is resource-intensive, requiring several hours for the largest CNN on NVidia V100 GPUs (hosted on the Cyclone supercomputer at the Cyprus Institute), the trained model is computationally efficient during inference. Once trained, the model can predict the internal temperature field within seconds per sample, making it suitable for real-time or near-real-time applications using standard hardware. The computational cost of training is largely dependent on the size and dimensionality of the training dataset, as well as the complexity of the model architecture. In contrast, the application of the model for prediction requires minimal resources, underscoring its practicality for use in various scenarios, including medical diagnostics and material testing.

### 2.4. Evaluation Metrics

In our evaluation, we utilize several metrics to assess the performance of the 3D convolutional neural network (CNN) in reconstructing the temperature field, with a particular focus on both the full 3D temperature distribution and the hot-spot regions. The metrics include the Structural Similarity Index Measure (SSIM), Peak Signal-to-Noise Ratio (PSNR), Mean Squared Error (MSE), Normalized Mean Squared Error (NMSE), Mean Absolute Error (MAE), Dice Coefficient, and Intersection over Union (IoU).

**Figure 3:**
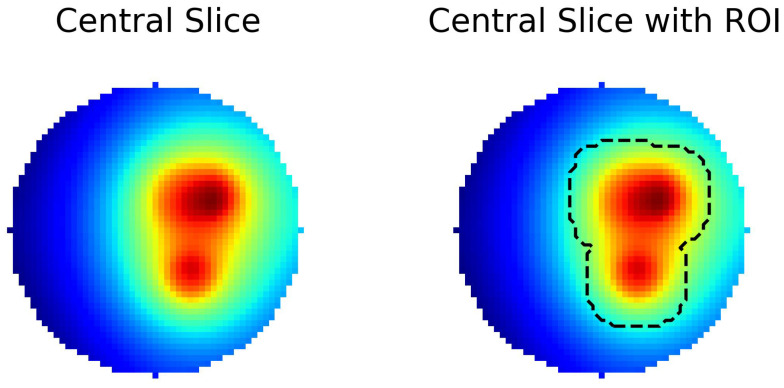
The ROI used for the local evaluation of the metrics. On the left, the central slice of a particular 3D temperature field is plotted. On the right, the ROI used for the local evaluation of the metrics is indicated with broken lines. The ROI highlights critical areas with significant temperature variations to assess reconstruction accuracy through localized metrics. This localized evaluation allows for a more detailed analysis of the predicted temperature fields, particularly in areas where the model’s performance is most relevant. Metrics such as the Structural Similarity Index (SSIM), Mean Squared Error (MSE), and Dice Coefficient were computed within the ROI to provide focused insights into the model’s strengths and limitations.

To ensure a localized evaluation of the network’s accuracy, we apply these metrics within a region of interest (ROI) corresponding to the enlarged source mask, in addition to assessing them across the entire 3D volume. The ROI is extracted by applying binary dilation to the original source mask, ensuring that it encompasses a sufficient area around the hotspot regions as shown for the middle slice in Figure 3. This dual evaluation allows us to examine the network’s overall reconstruction performance and its ability to accurately reconstruct critical areas of the image, specifically the regions influenced by the heat sources.

The region of interest (ROI) shown in Figure 3 was defined to include areas with significant temperature variations, such as hotspot regions, which are critical for evaluating the model’s reconstruction accuracy. This localized evaluation allows for a more detailed analysis of the predicted temperature fields, particularly in areas where the model’s performance is most relevant. Metrics such as the Structural Similarity Index (SSIM), Mean Squared Error (MSE), and Dice Coefficient were computed within the ROI to provide focused insights into the model’s strengths and limitations.

In the context of thermal tomography, the inverse problem is characterized by incomplete information, particularly when reconstructing internal temperature fields from surface measurements, while a more comprehensive error analysis under ideal conditions could be performed, such an approach risks overlooking the critical challenges associated with non-ideal scenarios. For small phantoms with ideal surface temperature readings, the error metrics would inevitably appear favorable. However, these results would fail to account for the inherent complexities and uncertainties of real-world applications. Instead, our focus is on compensating for missing information and recovering as much of the internal temperature field as possible, even under noisy and incomplete conditions. This approach reflects the practical realities of inverse problems and emphasizes the need for robust methods rather than idealized accuracy.

For each test sample, the SSIM quantifies the perceived similarity between the actual and predicted temperature distributions both across the entire 3D field and within the ROI, while the PSNR measures the reconstruction quality in terms of the ratio between the maximum possible intensity and the noise present. The MSE, NMSE, and MAE evaluate the pixel-wise differences between the predicted and true temperature fields. MSE penalizes larger errors more heavily, while NMSE, given by

$$\mathrm{NMSE}=\frac{\sum_{i=1}^{N}{\left(T_{\mathrm{true},i}-T_{\mathrm{pred},i}\right)}^{2}}{\sum_{i=1}^{N}T_{\mathrm{true},i}^{2}},$$

normalizes the MSE with the variance of the true values, providing a measure of the prediction error relative to the data’s overall variability. This makes NMSE particularly useful when comparing models across different datasets with varying magnitudes. The MAE provides a more robust metric against outliers by focusing on the average absolute error across all pixels.

In addition, the Dice Coefficient and IoU, commonly used in segmentation tasks, are calculated to assess the spatial overlap between the predicted and actual hot-spot regions by binarizing the images at half of the maximum temperature in the ROI. These metrics, combined, provide a comprehensive assessment of the network’s reconstruction accuracy, focusing on both the global temperature field and the localization of heat sources within the temperature distribution.

## 3. Results and Discussion

In this section, we present the evaluation of the 3D convolutional neural network (CNN) for the reconstruction of temperature fields. The network was tested on configurations containing between 1 and 6 heat sources, and we use several metrics to assess its performance, both globally and within the regions of interest (ROIs) surrounding the heat sources. The metrics include the Structural Similarity Index Measure (SSIM), Peak Signal-to-Noise Ratio (PSNR), Mean Squared Error (MSE), Mean Absolute Error (MAE), Dice Coefficient, and Intersection over Union (IoU).

### 3.1. Prediction with Ideal Conditions

Initially, the neural network was used employed at ideal conditions; i.e., the surface temperature used for the input sinograms was the theoretically exact one, as calculated by our model. The input to the network is a 3D array with dimensions 64×64×40, representing the surface sinograms. The results for the case of a small-sized phantom (diameter of 10 cm) and ideal conditions are summarized in Table 1.

**Figure 4:**
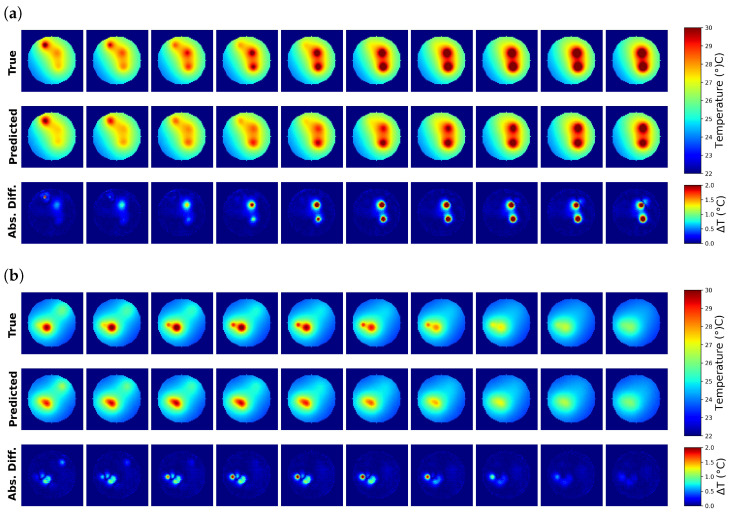
The 10 central slices along the z-axis for the ideal case of a 10 cm phantom. (**a**) One of the best predictions (MSE = 0.01). (**b**) One of the worst predictions (MSE = 1).

Figure 4 illustrates two cases of best and worst MSE for the case of three sources where the evaluation of MSE was performed in the ROI (see Figure 3).

There is no clear pattern in source position, size, or distribution that leads to a systematic decrease in the accuracy of the predictions, indicating that within this range, the inverse problem remains adequately tractable using our neural network.

For physical dimensions, such as a phantom diameter of 10 cm, the complete neural network architecture with residual blocks at each level proves to be highly effective. The residual blocks, designed to facilitate deeper networks and mitigate the vanishing gradient problem, allow for efficient feature extraction and hierarchical learning, capturing the intricate details of the temperature distribution within smaller phantoms. However, as the physical size of the domain increases, due to temperature diffusion, the impact of the high temperature of an inner hot-spot on the surface becomes less significant, especially at greater depths. In these cases, having a residual block at every level introduces unnecessary complexity and can impede the training process. The network becomes too deep and over-parameterized, leading to difficulties in minimizing the loss function due to the insufficient information from the surface.

To address this, a more simplified architecture is required. Either reducing the number of levels in the network or limiting the use of residual blocks (such as employing them only at critical locations, like the bottleneck) helps the model focus on extracting the most relevant features without over-complicating the learning process. This balance between network depth and the complexity of the problem ensures better training outcomes for larger physical dimensions, where less information is available from surface observations. Hence, adjusting the network’s architecture in response to the scale of the domain significantly improves the ability of the neural network to accurately reconstruct the temperature field. Using simplified neural networks, phantoms with a physical size of 20 cm, 40 cm, and 50 cm were additionally investigated leading to similar results.

### 3.2. Beyond Ideal Conditions

Until now, our analysis has focused on ideal conditions, neglecting any source of random or systematic errors. However, in practical thermal tomography applications, such errors are inevitable. Random errors may arise from inherent instabilities in infrared detectors, which can introduce noise into the temperature readings. Systematic errors, on the other hand, may result from environmental factors, such as fluctuating ambient temperatures or thermal gradients near the surface, which affect the surface temperature measurements. These variations can skew the input sinograms, leading to discrepancies in the predicted temperature fields. For instance, common issues in infrared thermography include drift due to detector aging or fluctuations in emissivity [7]. Such factors need to be carefully accounted for in real-world scenarios to ensure the reliability of the predictions.

**Figure 5:**
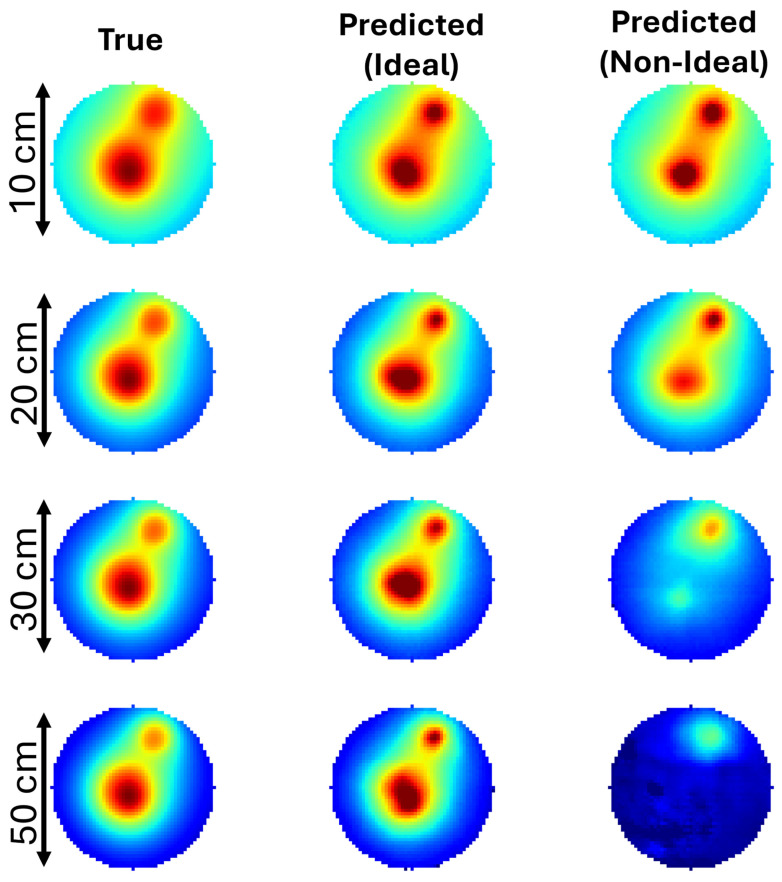
The application of the neural network for increasing physical size for ideal and non-ideal conditions (10% statistical noise and constant background temperature up to 0.1 K). The first column is the central slice obtained for various physical phantom sizes for a case with two hot-spots, one in the center and one near the surface. The middle column is the model prediction for ideal conditions (exact surface temperature). The third column is the prediction when 10% statistical noise and a constant background of 0.05 K are added.

We tested the applicability of our neural network across different physical sizes, with and without the addition of statistical and systematic errors. We included a 10% statistical noise and a background temperature which was different for each image taken by the detector, ranging between 0.05 K and 0.1 K. Under ideal surface temperature conditions, the models accurately predict the temperature distribution for phantoms of various sizes. When noise and background temperature are added to the input, the model’s ability to predict the temperature field becomes limited to regions closer to the surface, especially as the phantom size increases. This is qualitatively illustrated in Figure 5 using a single slice from the phantom. The collective statistical results for up to three hot-spots are shown in Table 2 for the case of the largest phantom (diameter of 50 cm).

**Figure 6:**
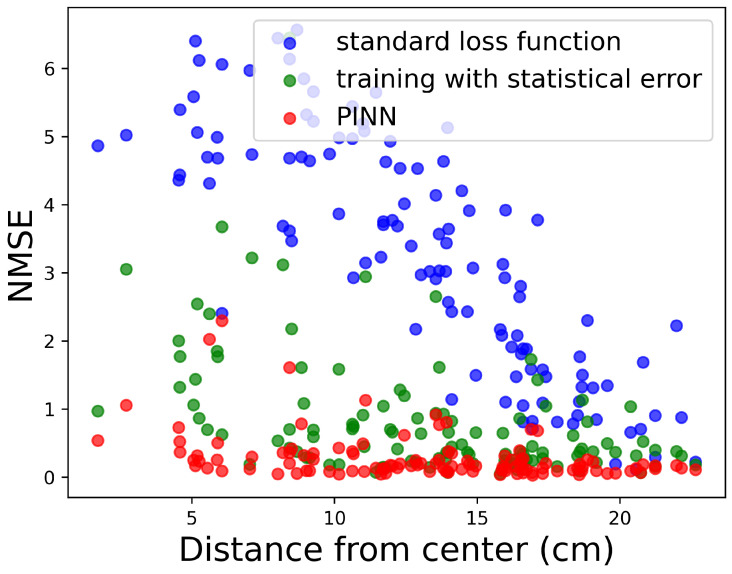
The value of the normalized NMSE for the prediction of the temperature field, originating from a single source located within a cylindrical domain of diameter 50 cm under non-ideal conditions (10% statistical noise and constant background temperature up to 0.1 K). Points in blue represent values with a standard loss function, points in green are the values after using noise during training, and points in red correspond to the addition of physics information (PINN).

In the presence of background noise, the accuracy of predicting the 3D temperature field diminishes as the physical size of the domain increases. The deeper regions of the phantom are more challenging to reconstruct due to the reduced information available from surface measurements, a feature illustrated in Figure 6 (green points), where the NMSE is plotted as a function of distance from the center of the domain for a domain with a radius up to 25 cm. For sources near the center of the domain, NMSE is maximized and decreases as the source location approaches the surface. This reduction in accuracy primarily limits the network’s ability to detect features deep within the material, allowing accurate predictions only near the surface.

## 4. Improving Prediction Using Noise and Physics-Informed Training

In the context of 3D thermal tomography, adding noise to the input during training has emerged as a common strategy for improving model performance, especially in challenging conditions involving noisy or incomplete data. This technique is employed to help the neural network generalize better and to simulate the non-ideal conditions that are often encountered in real-world applications. By training the neural network with input noise, the model learns to handle noisy data more effectively during inference, leading to improved robustness and prediction accuracy.

Recent studies have demonstrated the efficacy of this method across various domains of tomography and inverse problems. For instance, Habring and Holler [37] highlight neural-network-based approaches to regularization in imaging, which integrate noise during the training process to enhance model performance under real-world noise conditions. This practice is particularly useful in applications where measurement noise is unavoidable, such as in magnetic resonance imaging (MRI) and computed tomography (CT).

The rationale behind this approach is that introducing noise during training helps the network learn more generalized features, reducing its susceptibility to overfitting to idealized or clean datasets. This is similar to the concept of data augmentation, where variations are introduced to the training data to improve generalization. Notably, this strategy aligns with the findings of Gilton et al. [38], who demonstrated how noise-robust training can lead to superior performance in deep equilibrium architectures for inverse problems. Similarly, Mukherjee et al. [8] emphasized that adding noise to the training inputs allows learned reconstruction methods to achieve better convergence guarantees under noisy conditions.

In addition to adding noise, one can incorporate a physics-informed neural network (PINN) into the training process to improve the model’s robustness, especially in noisy environments. PINNs incorporate the governing physical laws, such as partial differential equations (PDEs), directly into the loss function during training, ensuring that the predicted solution adheres to these physical principles. This approach has been successfully applied in various fields, including heat transfer and fluid dynamics [35]. By embedding the physics of the heat equation directly into the loss function, we can constrain the neural network to not only minimize the prediction error but also to ensure that the predicted temperature field is consistent with the underlying physics of heat conduction and convection.

For our application, we use the heat equation as the governing physics, defined as:

$$\frac{\partial T}{\partial t}-\alpha \nabla^{2}T=f\left(x,y,z,t\right)$$

where *T* is the temperature field, α is the thermal diffusivity, and f(x,y,z,t) represents the heat sources. The corresponding loss function incorporates two components: a data-driven loss $L_{data}$, which minimizes the difference between predicted and true temperature fields, and a physics-informed loss $L_{physics}$, which ensures that the predicted temperature field satisfies the heat equation:

$$L_{total}=L_{data}+\lambda L_{physics}$$

where

$$L_{data}=\frac{1}{N}\sum_{i=1}^{N}{\left(T_{pred}\left(x_{i},y_{i},z_{i}\right)-T_{true}\left(x_{i},y_{i},z_{i}\right)\right)}^{2}$$

and

$$L_{physics}=\frac{1}{M}\sum_{j=1}^{M}{\left(\frac{\partial T_{pred}}{\partial t}-\alpha \nabla^{2}T_{pred}-f\left(x_{j},y_{j},z_{j},t_{j}\right)\right)}^{2}$$

$f(x_{j},y_{j},z_{j},t_{j})$ is the true source, *N* is the number of data points, *M* is the number of points used to evaluate the physics constraints, and λ is a weighting parameter that balances the contributions of the two loss terms. Furthermore, in our work we consider the steady state, i.e., $\frac{\partial T_{pred}}{\partial t}=0$.

**Figure 7:**
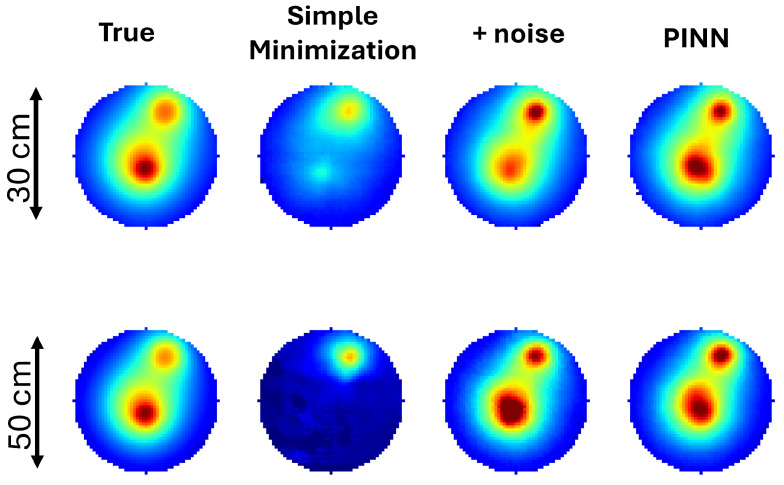
Comparison of predicted temperature fields under non-ideal conditions with and without noise addition during training and a physics-informed loss function.

In our study, noise statistically equivalent to that described in the previous sections was added to the input data during the training phase to simulate non-ideal conditions. This approach resulted in improved prediction accuracy compared to models trained solely on noise-free data. In addition, combining the addition of noise with the physics-informed approach further enhances the model predictive ability. Figure 7 demonstrates qualitatively the improvement when noise and physics information are used in the training process. Noise alone can restore most of the lost information and the combination with a PINN furthers improve the prediction as is quantitatively illustrated in Table 3 and Figure 6. Table 3 conatins the collected statics for selected metrics, while Figure 6 examines the dependence of the NMSE to its distance from the phantom’s center, demonstrating that the loss of information is greatly reduced, particularly when both noise and a PINN are used.

## 5. Conclusions and Future Outlook

In this work, we developed and tested a 3D convolutional neural network (CNN) for reconstructing the internal temperature field from surface temperature sinograms. The network was evaluated under both ideal and non-ideal conditions, with random and systematic errors introduced to simulate realistic infrared detector inaccuracies and environmental conditions. Our results show that, for small physical dimensions (e.g., phantoms with a diameter of 10 cm), the full neural network with residual blocks at every level is effective in accurately predicting the internal temperature field. Under ideal conditions, no systematic trend is observed. The CNN captures the true temperature field with high precision, which is an expected outcome and aligns with findings from previous studies.

However, in the absence of ideal conditions and as the physical size increases, the network’s prediction ability becomes restricted to regions near the surface due to the lack of sufficient information about the internal structures. Trends in the validity of the reconstructed internal temperature field become apparent, indicating that the model performs better when fewer hotspots are present, as simpler temperature distributions provide more direct and informative surface data. As the number of hotspots increases, the complexity of the internal structure grows, leading to reduced accuracy in the reconstructed temperature fields. This decrease in performance reflects the inherent challenge of resolving finer details in more complex temperature distributions.

To mitigate this limitation, we introduced noise during the training process in combination with physics-informed neural networks (PINNs), incorporating the principles of heat conduction directly into the loss function. By ensuring that the predicted temperature field adheres to the heat equation, this approach improved the network’s performance, especially under non-ideal conditions. The PINN-enhanced model showed better accuracy in predicting temperature fields, even in the presence of noise and for larger physical domains. These advancements demonstrate the potential of combining deep learning with physical laws to improve the robustness and applicability of thermal tomography.

For future research, it is essential to acknowledge that the current model assumes constant thermal conductivity and simple geometries and relies solely on synthetic data for evaluation. While synthetic data provide a controlled environment to systematically assess the framework’s performance, they do not fully capture the complexities and variability of real-world scenarios. To address this, we aim to validate the proposed method using experimental datasets derived from CT scans of anatomical structures, such as the human wrist. These scans would allow us to incorporate variable thermal conductivity, providing a more accurate representation of the internal thermal environment. The software model would translate the CT scan data into a finite element model (FEM), where the actual material properties can be assigned to different regions of the domain based on the anatomical structure. This approach would lead to more realistic simulations and improved diagnostic capabilities in thermal imaging applications. Finally, we plan to investigate cases where the hot-spots’ temperature differences with the surrounding material are even lower, ideally 1–2 °C, to further test the robustness of the framework.

It is important to note that this work primarily focuses on reconstructing near-surface temperature fields, where surface temperature data provide sufficient information for accurate predictions. While the incorporation of PINNs and noise-aware training has improved the method’s robustness for larger domains, the prediction accuracy decreases with depth due to the inherent limitations of inverse problems. Addressing deep tissue analysis or large-scale material testing is beyond the scope of this study and represents an area for future research.

## Figures and Tables

**Table 1:** Performance metrics for different numbers of hot-spots (rounded to two significant figures). This is for the case of ideal conditions and a 10 cm phantom.

Metric	No. of Hot-Spots					
	1	2	3	4	5	6
SSIM	0.95 ± 0.07	0.98 ± 0.02	0.98 ± 0.01	0.99 ± 0.01	0.98 ± 0.01	0.98 ± 0.01
MSE	0.0061 ± 0.0064	0.011 ± 0.0095	0.019 ± 0.017	0.028 ± 0.018	0.046 ± 0.027	0.060 ± 0.033
PSNR	35 ± 4	36 ± 3	35 ± 3	36 ± 2	34 ± 2	33 ± 2
MAE	0.034 ± 0.0047	0.046 ± 0.0090	0.057 ± 0.011	0.073 ± 0.013	0.093 ± 0.017	0.11 ± 0.023
NMSE	0.0340 ± 0.0414	0.0216 ± 0.0122	0.0202 ± 0.0130	0.0180 ± 0.0092	0.0234 ± 0.0110	0.0256 ± 0.0123
Dice	0.83 ± 0.23	0.86 ± 0.14	0.83 ± 0.25	0.85 ± 0.19	0.84 ± 0.19	0.83 ± 0.19
IoU	0.75 ± 0.23	0.78 ± 0.17	0.77 ± 0.26	0.78 ± 0.21	0.76 ± 0.21	0.74 ± 0.22
ROI Metrics						
SSIM (ROI)	0.91 ± 0.08	0.95 ± 0.03	0.96 ± 0.02	0.97 ± 0.02	0.96 ± 0.02	0.96 ± 0.02
MSE (ROI)	0.13 ± 0.29	0.10 ± 0.14	0.10 ± 0.13	0.11 ± 0.10	0.16 ± 0.12	0.17 ± 0.13
PSNR (ROI)	23 ± 6	27 ± 4	29 ± 4	30 ± 3	29 ± 3	30 ± 3
MAE (ROI)	0.15 ± 0.11	0.15 ± 0.09	0.15 ± 0.06	0.16 ± 0.05	0.20 ± 0.06	0.21 ± 0.06
NMSE (ROI)	0.1675 ± 0.2087	0.0965 ± 0.0707	0.0768 ± 0.0626	0.0538 ± 0.0408	0.0657 ± 0.0384	0.0590 ± 0.0311
Dice (ROI)	0.83 ± 0.23	0.87 ± 0.14	0.84 ± 0.25	0.86 ± 0.19	0.85 ± 0.19	0.83 ± 0.19
IoU (ROI)	0.76 ± 0.24	0.79 ± 0.17	0.77 ± 0.26	0.79 ± 0.21	0.77 ± 0.22	0.75 ± 0.22

**Table 2:** SSIM and NMSE transition from ideal to noisy conditions for different numbers of hotspots (diameter = 50 cm).

Metric	No. of Hot-Spots (Ideal → Noisy)		
	1	2	3
SSIM	0.95 ± 0.056 → 0.45 ± 0.19	0.98 ± 0.017 → 0.49 ± 0.17	0.99 ± 0.0065 → 0.61 ± 0.14
NMSE	0.038 ± 0.038 → 0.83 ± 0.48	0.030 ± 0.018 → 0.79 ± 0.41	0.027 ± 0.020 → 0.69 ± 0.34
ROI Metrics			
SSIM (ROI)	0.93 ± 0.058 → 0.37 ± 0.31	0.95 ± 0.028 → 0.45 ± 0.22	0.96 ± 0.017 → 0.56 ± 0.17
NMSE (ROI)	0.15 ± 0.16 → 2.8 ± 1.9	0.10 ± 0.076 → 2.1 ± 1.4	0.079 ± 0.058 → 1.6 ± 0.97

**Table 3:** SSIM and NMSE metrics for different numbers of hot-spots under various training conditions (non-ideal conditions and diameter = 50 cm).

Metric	No. of Hot-Spots					
	1	2	3	4	5	6
Without Noise or PINN						
SSIM	0.43 ± 0.17	0.38 ± 0.15	0.38 ± 0.15	0.49 ± 0.14	0.55 ± 0.12	0.60 ± 0.11
NMSE	0.95 ± 0.43	1.09 ± 0.44	1.22 ± 0.49	0.97 ± 0.43	0.95 ± 0.40	0.84 ± 0.34
SSIM (ROI)	0.28 ± 0.26	0.31 ± 0.20	0.35 ± 0.19	0.49 ± 0.18	0.52 ± 0.14	0.57 ± 0.12
NMSE (ROI)	3.23 ± 1.73	2.87 ± 1.56	2.81 ± 1.45	1.96 ± 1.16	1.87 ± 1.01	1.55 ± 0.76
Noise Added During Training						
SSIM	0.65 ± 0.19	0.82 ± 0.13	0.92 ± 0.07	0.95 ± 0.04	0.95 ± 0.02	0.96 ± 0.01
NMSE	0.30 ± 0.30	0.16 ± 0.16	0.08 ± 0.06	0.06 ± 0.03	0.06 ± 0.03	0.06 ± 0.03
SSIM (ROI)	0.72 ± 0.21	0.82 ± 0.13	0.89 ± 0.05	0.91 ± 0.04	0.91 ± 0.04	0.91 ± 0.03
NMSE (ROI)	0.82 ± 0.93	0.42 ± 0.53	0.19 ± 0.14	0.14 ± 0.09	0.14 ± 0.07	0.13 ± 0.06
With Noise During Training and PINN						
SSIM	0.91 ± 0.09	0.96 ± 0.02	0.97 ± 0.01	0.97 ± 0.01	0.97 ± 0.01	0.97 ± 0.01
NMSE	0.08 ± 0.09	0.05 ± 0.03	0.05 ± 0.03	0.04 ± 0.02	0.05 ± 0.02	0.06 ± 0.02
SSIM (ROI)	0.87 ± 0.11	0.91 ± 0.05	0.93 ± 0.03	0.93 ± 0.03	0.92 ± 0.02	0.92 ± 0.03
NMSE (ROI)	0.29 ± 0.35	0.17 ± 0.11	0.13 ± 0.09	0.11 ± 0.07	0.13 ± 0.06	0.12 ± 0.05

## Data Availability

The dataset used for training the neural network (composed of the training, validation and testing sets) is available at https://figshare.com/articles/dataset/3D_dataset_for_thermal_tomography/27611766 (accessed on 5 November 2024).
